# Transient receptor potential ankyrin 1 ion channel expressed by the Edinger-Westphal nucleus contributes to stress adaptation in murine model of posttraumatic stress disorder

**DOI:** 10.3389/fcell.2022.1059073

**Published:** 2022-12-06

**Authors:** János Konkoly, Viktória Kormos, Balázs Gaszner, Pedro Correia, Gergely Berta, Tünde Biró-Sütő, Dóra Zelena, Erika Pintér

**Affiliations:** ^1^ Department of Pharmacology and Pharmacotherapy, University of Pécs, Medical School, Pécs, Hungary; ^2^ Department of Anatomy, University of Pécs, Medical School, Pécs, Hungary; ^3^ Department of Physiology, University of Pécs, Medical School, Pécs, Hungary; ^4^ Department of Medical Biology, University of Pécs, Medical School, Pécs, Hungary; ^5^ Signal Transduction Research Group, János Szentágothai Research Centre, Pécs, Hungary

**Keywords:** TRPA1, PTSD, Edinger-Westphal nucleus, single prolonged stress, urocortin 1, stress adaptation

## Abstract

The centrally projecting Edinger-Westphal nucleus (EWcp) is involved in stress adaptation. Transient receptor potential ankyrin 1 (TRPA1) mRNA was previously shown to be expressed abundantly in mouse and human EWcp urocortin 1 (UCN1) positive neurons and reacted to chronic stress. Since UCN1 neurons are deeply implicated in stress-related disorders, we hypothesized that TRPA1/UCN1 neurons are also affected in posttraumatic stress disorder (PTSD). We examined male *Trpa1* wild type (WT) and gene-deficient (KO) mice in the single prolonged stress (SPS) model of PTSD. Two weeks later the behavioral changes were monitored by forced swim test (FST) and restraint. The *Trpa1* and *Ucn1* mRNA expression and the UCN1 peptide content were assessed by RNAscope *in situ* hybridization technique combined with immunofluorescence labeling in the EWcp. SPS-induced immobility was lower in *Trpa1* KO compared to WT animals, both in the FST and restraint, corresponding to diminished depression-like behavior. The copy number of *Trpa1* mRNA decreased significantly in EWcp of WT animals in response to SPS. Higher basal *Ucn1* mRNA expression was observed in the EWcp of KO animals, that was not affected by SPS exposure. EWcp neurons of WT animals responded to SPS with substantially increased amount of UCN1 peptide content compared to control animals, whereas such changes were not observable in KO mice. The decreased *Trpa1* mRNA expression in the SPS model of PTSD associated with increased neuronal UCN1 peptide content suggests that this cation channel might be involved in the regulation of stress adaptation and may contribute to the pathomechanism of PTSD.

## 1 Introduction

Transient receptor potential ankyrin 1 (TRPA1) is a non-selective cation channel expressed in the primary sensory neurons of the dorsal root, vagal and trigeminal ganglia (Talavera et al., 2020). In the peripheral nervous system it is involved in pain sensation, inflammatory and immune responses (Julius, 2013; Moore et al., 2018; Souza Monteiro de Araujo et al., 2020). Limited knowledge is available about its location and role in the central nervous system. Its expression has already been confirmed by our research group in certain stress-related brain areas, including olfactory bulb, piriform cortex (Konkoly et al., 2021), hypothalamus (Olah et al., 2021) and dorsal raphe nucleus (DR) (Milicic et al., unpublished). The highest level of *Trpa1* mRNA expression was found in the urocortinergic neurons of the centrally projecting Edinger-Westphal nucleus (EWcp) in mice (Kormos et al., 2022). Most recently we found that the TRPA1 ion channel shows functional activity in patch clamp recordings on acute mouse EWcp slices (Al-Omari et al., unpublished). Our previous study revealed that *Trpa1* is downregulated in the EWcp both upon chronic variable mild stress (CVMS) in mice and in humans who died by suicide. Moreover, altered stress adaptation response was observed in the absence of TRPA1 in mice (Kormos et al., 2022). These findings suggested that TRPA1 expressed by the urocortinergic EWcp neurons might contribute to stress response. Considering that a disturbed stress adaptation is characteristic for posttraumatic stress disorder (PTSD) (Perry and Pollard, 1998; Yehuda, 1998; Kasckow et al., 2001; Rohleder et al., 2010; Deppermann et al., 2014; Dunlop and Wong, 2019), we investigated the role of TRPA1 in this psychopathology.

PTSD is a mental health condition triggered by intense physical or emotional trauma (*e.g.:* accident, childhood abuse, sexual violence, physical assault, combat exposure and other life-threatening events). Flashbacks, severe anxiety, and nightmares associated with intrusive memories, negative changes in mood and thinking, and altered physical and emotional reactions are all possible symptoms (Auxéméry, 2018). The most important brain areas being involved in the pathogenesis of PTSD are the prefrontal cortex (PFC), amygdala, and hippocampus (Miller et al., 2018; Harnett et al., 2020; Kamiya and Abe, 2020). The EWcp and its urocortinergic neurons have not been studied in relation to PTSD, yet.

Our research team has been investigating the role of the EWcp in stress adaptation response and mood control (Kozicz et al., 2001; Gaszner and Kozicz, 2003; Gaszner et al., 2004; Gaszner et al., 2007, 2012; Rouwette et al., 2011; Kormos and Gaszner, 2013; Kormos et al., 2022; Ujvári et al., 2022). EWcp expresses urocortin 1 (UCN1) neuropeptide, which is a member of the corticotropin-releasing hormone (CRH) family. It binds to the CRH receptor 1 (CRHR1) and 2 (CRHR2) (Kozicz, 2007), with a 40-fold higher affinity towards CRHR2 than CRH itself (Vaughan et al., 1995; Deussing and Chen, 2018). Interestingly, direct connections have been identified between the EWcp and the most relevant PTSD-related limbic brain areas (PFC, amygdala and hippocampus). For instance, the EWcp sends fibers to the PFC and amygdala (Zuniga and Ryabinin, 2020; Priest et al., 2021; Topilko et al., 2022). EWcp cells receive direct synaptic inputs from hippocampal pyramidal cells that carry serotonin receptor 2c (Li et al., 2018). Peptidergic EWcp cells send afferent projections to GABAergic parvalbumin-containing interneurons in the medial PFC (Bale et al., 2002). Importantly, CRH receptors were found in all three areas (PFC, amygdala and hippocampus) implicated in PTSD (Deussing and Chen, 2018).

Here we aimed to test whether TRPA1 on the EWcp/UCN1 neurons is important in stress adaptation to PTSD. We hypothesized that altered TRPA1 and UCN1 dynamics in the EWcp contribute to the behavioral anomalies induced by PTSD. To test this hypothesis, we used the single prolonged stress (SPS) model of PTSD and examined the behavioral alterations 2 weeks later. To support possible causative role, *Trpa1* KO mice strain was also used.

## 2 Materials and methods

### 2.1 Animals

3–4 months-old male *Trpa1* ^+/+^ (wild type, WT) and *Trpa1* ^−/−^ (knockout, KO) mice were used. The original breeding pairs were acquired from Prof. P. Geppetti, University of Florence, Italy), originally generated by Bautista and co-workers (Bautista et al., 2006). Mice were generated and characterized as described earlier (Meza et al., 2006). Animals were bred on a C57BL/6J background and crossed back after 10 generations. The genotype of offspring for the *Trpa1* gene was verified by PCR (sequences of primers: ASM2: ATC ACC TAC CAG TAA GTT CAT; ASP2: AGC TGC ATG TGT GAA TTA AAT).

Animals were housed in the animal facility of the Department of Pharmacology and Pharmacotherapy, University of Pécs in a temperature and humidity controlled 12 h light–dark cycle environment (lights on at 6 a.m.) in standard polycarbonate cages (365 mm × 207 mm × 144 mm). *Ad libitum* standard rodent chow and tap water were provided for the animals. Four to six mice were housed in one cage.

During the experiments all efforts were provided to reduce the number of animals used and their suffering. All procedures applied in this protocol were approved by the Animal Welfare Committee at Pécs University and National Scientific Ethical Committee on Animal Experimentation in Hungary (permission No: BA02/2000/33/2018) in agreement with the directive of the European Communities Council in 1986, and with the Law of XXCIII, in 1998, on Animal Care and Use in Hungary.

### 2.2 Experimental design

**FIGURE 1:**
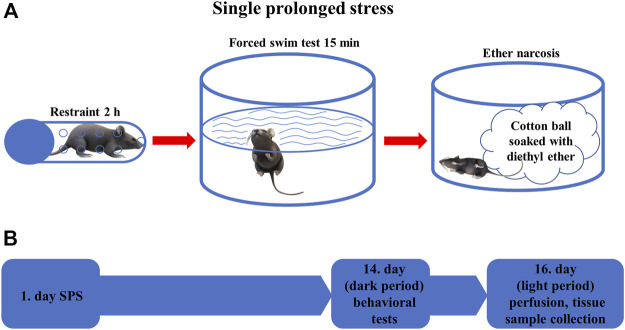
**(A)** Schematic representation of the single prolonged stress (SPS) experiment. The animals were restrained for 2 h, then forced to swim for 15 min before being exposed to diethyl ether narcosis until loss of consciousness **(B)** Time course of the behavioral experiments. On the first day, SPS was performed in the stressed groups of animals, after 2 weeks behavioral tests were performed in all animals. On the 16th day, mice were sacrificed, and tissue samples were collected for morphological analysis.

Animals were divided into four experimental groups: *Trpa1* KO (*n* = 12) and WT (*n* = 9) mice were exposed to SPS model of PTSD, while another set of *Trpa1* KO (*n* = 10) and WT (n = 10) mice were used as non-stressed controls. Animals were kept undisturbed in their home cages for 2 weeks after the SPS exposure then they were examined in the behavioral tests (forced swim test (FST), restraint). SPS experiments and the related behavioral tests were carried out during the early dark phase (i.e.: active phase of mice) between 18 and 22 h. To exclude the effect of acute stress caused by the behavioral tests, all animals were sacrificed for morphological studies 36 h after the last test (Figure 1).

### 2.3 Single prolonged stress

The SPS protocol was conducted as described earlier (Török et al., 2019). Briefly, each stressed animal was restrained for 2 h in a 50 ml conical tube closed with a screw on the top and having ventilation holes on the wall. Immediately after the restraint stress, the mice were forced to swim for 15 min in a 2 L Pyrex^®^ graduated beaker filled with water to a depth of about 20 cm (1.5 L) at 24 ± 1°C. Then the animals were dried with a towel and returned to their own cages for 15 min. Finally, each mouse was exposed to diethyl ether until loss of consciousness. At the end of the SPS, mice were placed in new cages with fresh bedding. The translational value of SPS is based on the phenomenon that most human PTSD is triggered by combined stressors (Lisieski et al., 2018) and lead to elevated fear response and impaired fear extinction (Yamamoto et al., 2008; Ganon-Elazar and Akirav, 2012; Perrine et al., 2016).

#### 2.3.1 Behavioral experiments

Behavioral experiments were carried out 14 days after the SPS protocol. The behavioral responses of mice were video recorded and later scored by computer-based event-recorder software (Solomon coder https://solomon.andraspeter.com) by an experimenter blinded to the treatment groups. Both the duration (in percentage) and frequency of all behavioral parameters were registered upon these tests.

#### 2.3.2 Restraint

We applied the restraint stress again according to the protocol described above with the sole important difference that this time it only lasted for 15 min. We investigated the time spent immobility. Higher immobility time is characteristic for PTSD-like behavior (Török et al., 2019).

#### 2.3.3 Forced swim test

After 1 h rest, each animal was exposed to FST similarly to described above for a total duration of 6 min. The following behavioral parameters were investigated: floating (no obvious activity or balancing only with one of the hind paws) as immobility; swimming (movements to emerge the head from the water using both hind paws) and struggling behavior (intense motion with all paws to escape from the beaker) as mobility. Immobility reflects passive coping (Sabban et al., 2015; Kormos et al., 2022) and is considered a sign of PTSD (Török et al., 2019).

### 2.4 Perfusion, tissue collection

Thirty-six hours after the behavioral experiments all animals were deeply anesthetized by intraperitoneal urethane injection (2.4 g/kg) and transcardially perfused with 20 ml of ice-cold 0.1 M phosphate-buffered saline (PBS, pH: 7.4) followed by 150 ml 4% paraformaldehyde (PFA) solution in Millonig buffer (pH 7.4) for 15 min. After this procedure, the brains were removed, and collected into PFA for 72 h postfixation at 4°C. The brains were coronally sectioned using a Leica VT1000 S vibratome (Leica Biosystems, Wetzlar, Germany), three series of 30 µm sections were collected and stored in antifreeze solution (20% ethylene glycol, 30% glycerol and 0.1 M sodium-phosphate buffer) at −20°C. Sections containing the EWcp (from Bregma -2.92 mm to -4.04 mm according to Paxinos and Franklin (Paxinos and Franklin, 2001)) were selected for morphological studies.

### 2.5 RNAscope *in situ* hybridization (ISH) combined with immunofluorescence

The RNAscope ISH was performed on two different series of coronal EWcp sections to detect the *Ucn1* and *Trpa1* mRNA expression. RNAscope Multiplex Fluorescent Reagent Kit v.2 (Advanced Cell Diagnostics, Newark, CA, United States ) was used according to the protocol described earlier by our research group (Konkoly et al., 2021). Briefly, after the tissue pretreatment, samples were hybridized with the probe specific to mouse *Trpa1* (ACD, Cat. No. 400,211-C2) and *Ucn1* (ACD, Cat. No. 466,261) mRNA. In our KO animals the major part of exon 23 was replaced, thus, a modified *Trpa1* mRNA and mutated TRPA1 protein is expressed in KO mice with loss of function. However, the commercially available RNAscope probe hybridizes with the non-deleted mRNA sequence of the non-functioning protein, the RNAscope gives positive signal in knockout mice also.

Signal amplification and channel development was carried out according to the manual. In case of RNAscope 3-plex mouse positive control probes (ACD; Cat. No. 320,881, Advanced Cell Diagnostics, Newark, CA, United States ), specific to RNA polymerase II subunit A mRNA (*Polr2a* (fluorescein)), peptidylprolyl isomerase B mRNA (*Ppib* (cyanine 3, Cy3)) and ubiquitin C mRNA (*Ubc* (cyanine 5, Cy5)), and 3-plex negative control probes (ACD; Cat. No. 320,871), specific to bacterial D-box binding PAR BZIP transcription factor (*dabP*) mRNA, were also used as technical controls. That series, where the *Trpa1* was the target, ISH was combined with immunofluorescence to measure the UCN1 peptide content of the neurons in the EWcp (Kormos et al., 2022). Briefly, after channel development of the RNAscope ISH, sections were washed for 2 × 15 min in PBS, incubated overnight at room temperature (RT) with recombinant anti-urocortin 1 antibody (Abcam Cat. No. ab283503) diluted (1:10.000) in PBS with 2% normal donkey serum. Sections were washed for 2 × 15 min in PBS and incubated in Alexa Fluor 488-conjugated donkey anti-rabbit secondary antibody (Jackson ImmunoResearch Europe Ltd., Cambridgeshire, UK; Cat. No. 711-545-152), diluted to 1:500 in 1 × PBS with 2% normal donkey serum for 3 h at RT. After rinses, we applied 4′,6-diamidino-2-phenylindole (DAPI (Cat. No. 323,108, Advanced Cell Diagnostics, Newark, CA)) to visualize cell nuclei and sections were mounted with ProLong Diamond Antifade Mountant (Thermo Fisher Scientific, Waltham, MA, United States ) for confocal microscopy.

### 2.6 Microscopy, digital imaging, and morphometry

For imaging we used the Olympus Fluoview FV-1000 laser scanning confocal microscope and FluoView FV-1000S-IX81 image acquisition software system (Olympus Europa, Hamburg, Germany). Digital images were obtained by sequential scanning in analogue mode for the respective fluorophores to avoid false positive signal due to the slightly overlapping emission spectra and to detect reliably quantifiable fluorescent signal. The confocal aperture was set to 80 μm, and the analogue sequential scanning was performed using a ×60 objective lens (NA: 1.35). An optical thickness of 3.5 µm was calculated by the software and the resolution was set to 1,024 × 1,024 pixel. The excitation time was set to 4 µs per pixel. DAPI was excited at 405 nm, Fluorescein as well as Alexa Fluor 488 at 488 nm and Cy3 at 543 nm. To visualize the different targets, we used the following virtual colors for the fluorescent signals: blue for DAPI, red for Cyanine 3, green for Fluorescein and Alexa 488.

The morphometry was performed using ImageJ software (version 1.52a, NIH, United States ) on non-edited pictures. In case of the *Ucn1* mRNA and UCN1 peptide the intensity of the fluorescence was measured in 5-10 cell bodies for *Ucn1*/UCN1 using four non-edited images of the corresponding channel. The region of interest was manually determined at cytoplasmic areas of neurons. The signal density was measured and corrected for the background signal. The specific signal density (SSD) was expressed in arbitrary units (au). The average of the SSD of 5-10 neurons was quantified in four sections. The average of these four values represented the SSD value of one mouse.

In case of the *Trpa1* mRNA signal we manually counted the number of copies per cell in 5-10 neurons of a section, in four representative sections per animal. Finally, these values were averaged as described above and subjected to the statistical assessment.

### 2.7 Statistical analysis

All statistical analyses were carried out applying Statistica 13.5.0 software. Data are represented as mean ± SEM. Datasets were tested for normal distribution and for homogeneity of variance. The comparison between mRNA expression of control and stressed WT animals was performed by paired sample *t*-test. In the further experiments, the main effects were studied by two-way analysis of variance (ANOVA, factors SPS and genotype) followed by Tukey’s *post hoc* tests. If the *p*-value was lower than 0.05, it was considered statistically significant.

## 3 Results

### 3.1 TRPA1 KO mice show blunted SPS-induced immobility in the restraint stress compared to the stressed WT animals

**FIGURE 2:**
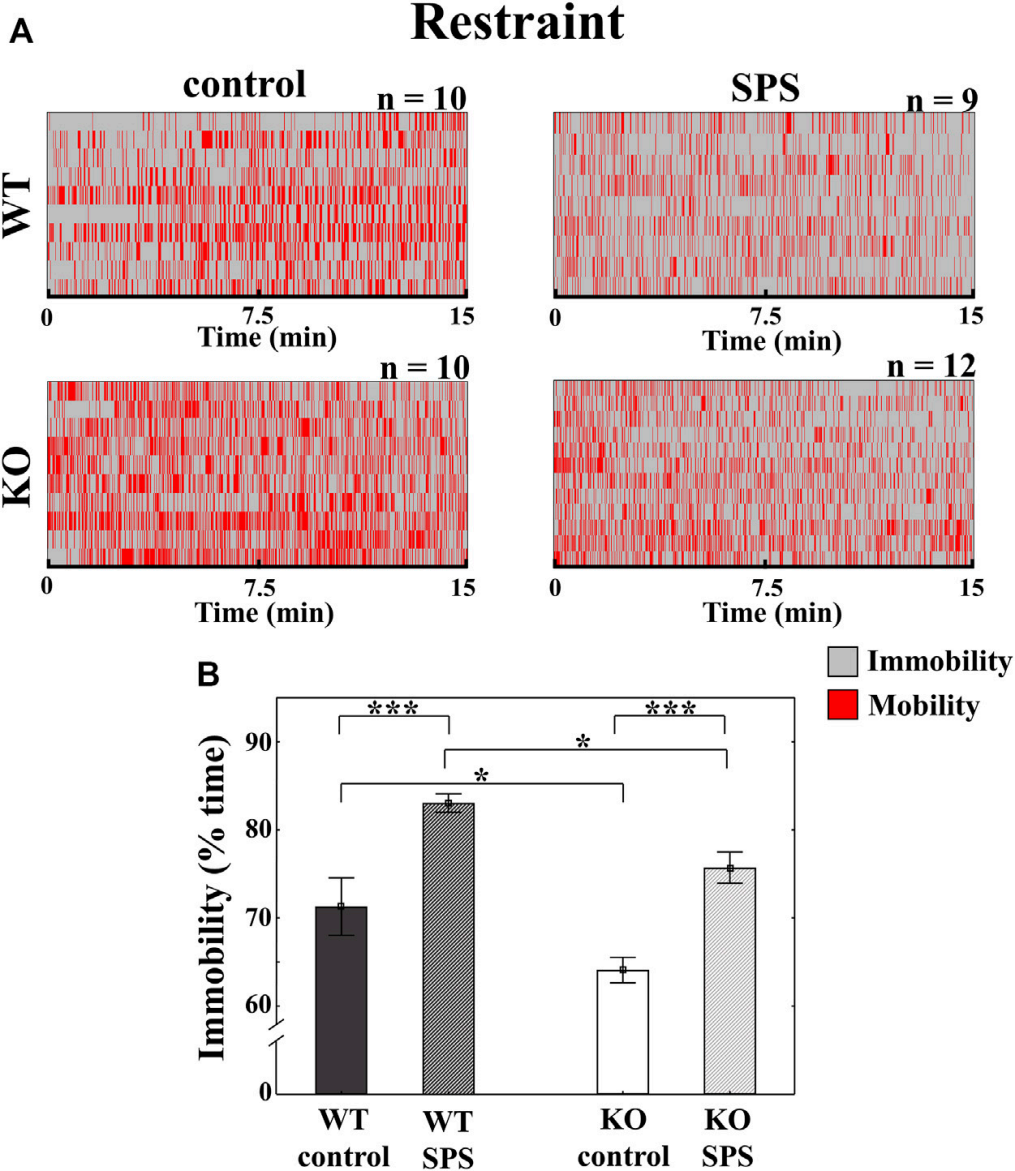
Alterations of the behavioral parameters in *Trpa1* wild type (WT) and knockout (KO) mice during the restraint stress. Individual values are represented on a Gantt diagram **(A).** Columns represent the time of immobility **(B)** (two-way ANOVA followed by Tukey’s *post-hoc* test; *p < 0.05 and ***p < 0.001; *n* = 9–12/group). SPS: single prolonged stress.

In restraint stress, we detected enhanced immobility in both stressed groups compared to the control, non-stressed mice with a significant main effect of stress (F_SPS_ (1.36) = 30.34, *p* < 0.01). The determinant influence of the genotype (F_genotype_ (1.36) = 11.81, *p* < 0.01) meant that the degree of immobility was significantly lower in both groups of KO mice in comparison with the related WT animals without interaction between stress and genotype (Figure 2).

#### 3.1.1 TRPA1 KO mice do not show SPS-induced immobility in forced swim test

**FIGURE 3:**
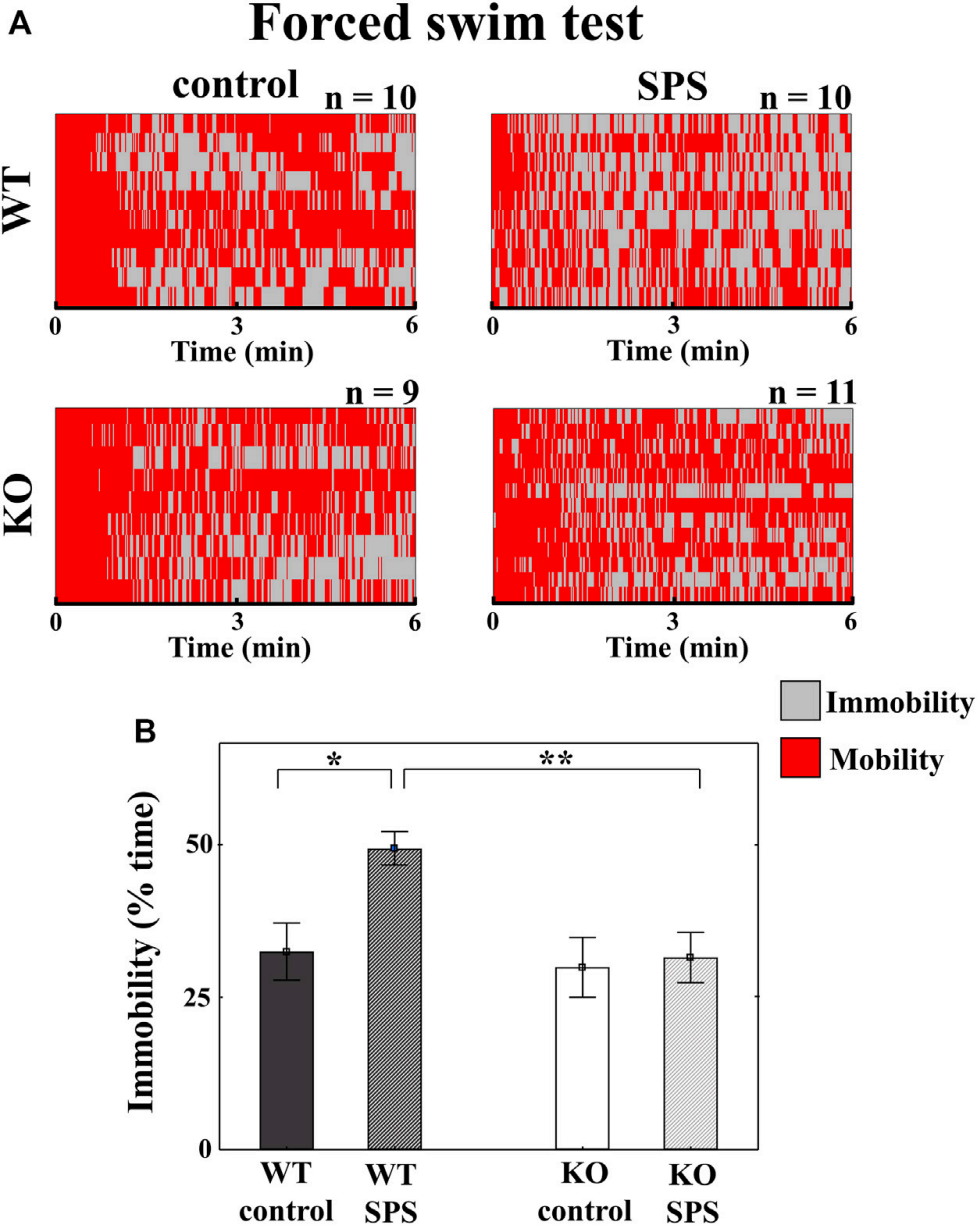
Changes in the behavioral parameters in *Trpa1* wild type (WT) and knockout (KO) mice during the forced swim test. Individual values are represented on a Gantt diagram **(A)**. Columns depict the duration of immobility **(B)** (two-way ANOVA followed by Tukey’s *post-hoc* test; *p < 0.05, **p < 0.01, and ***p < 0.001; n = 9–12/group). SPS: single prolonged stress.

During the FST, stressed WT mice showed significantly increased immobility compared to their control counterparts, however such differences were not detectable in case of KO animals with a significant main effect of the genotype in ANOVA (F_genotype_ (1.37) = 5.61, *p* < 0.03), and strong tendency in the main effect of stress (F_SPS_ (1.37) = 4.07, *p* < 0.06), but without interaction between stress and genotype (Figure 3).

### 3.2 *Trpa1* mRNA expression is downregulated in the urocortinergic neurons of the EWcp upon SPS

**FIGURE 4:**
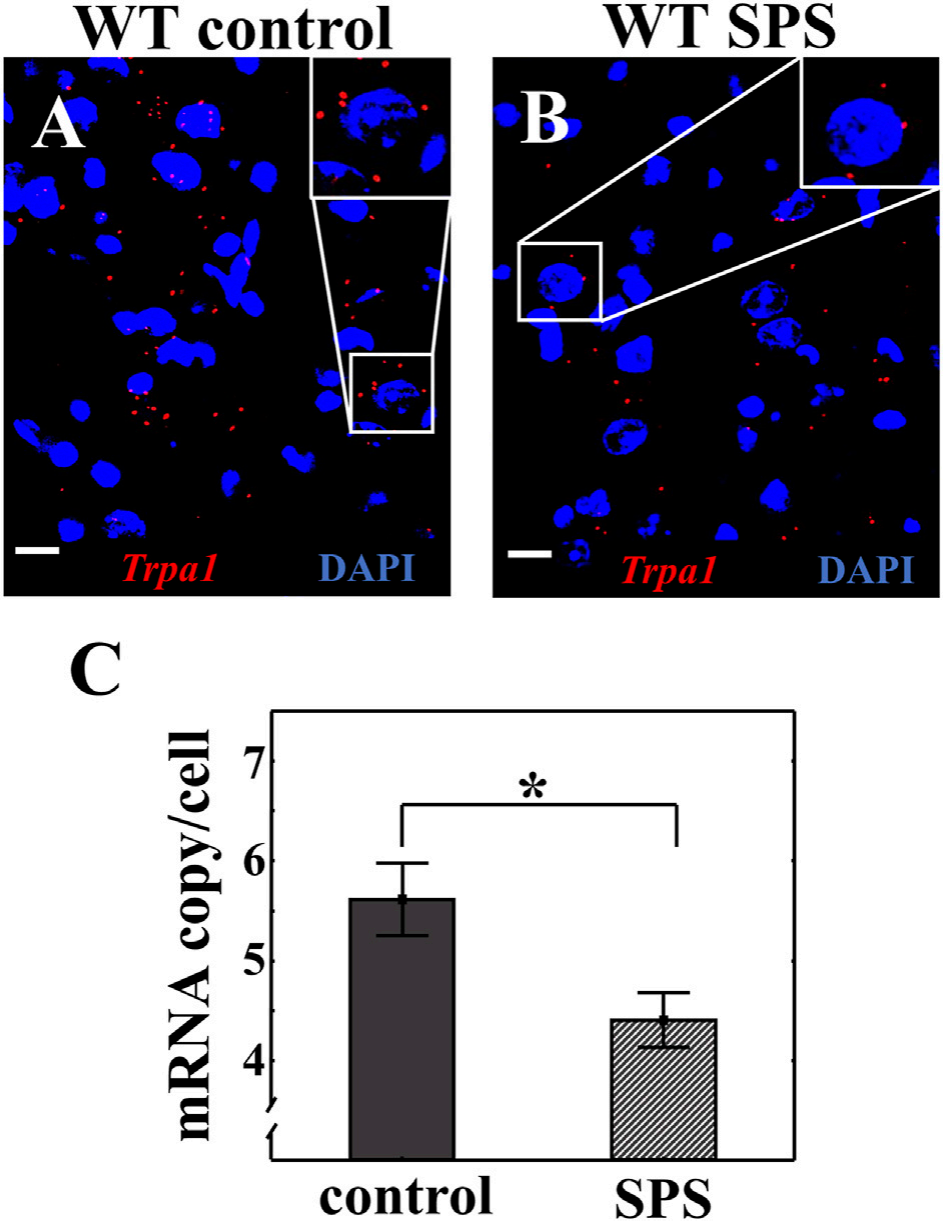
Effect of single prolonged stress (SPS) on the *Trpa1* expression in the centrally projecting Edinger-Westphal nucleus (EWcp). Representative image of the *Trpa1* mRNA expression in the EWcp neurons of wild type (WT) mice **(A)**. Effect of SPS on the *Trpa1* mRNA expression **(B).** Red dots represent *Trpa1* mRNA copies while cell nuclei were counterstained with 4′,6-diamidino-2-phenylindole (DAPI) (blue). Scale bars: 25 µm. Statistical analysis established significantly decreased amount of *Trpa1* mRNA in the EWcp after SPS (Student’s *t*-test; *p < 0.05; n = 7/groups) **(C)**.

RNAscope ISH was used to investigate the *Trpa1* expression in the EWcp combined with UCN1 immunofluorescent signal in WT animals. We have proven once again the colocalization of *Trpa1* transcripts with UCN1 peptide in the EWcp neurons (Suppl. Figure 1). We detected significantly lower number of *Trpa1* mRNA copies in the urocortinergic neurons of stressed animals compared to the controls (t_copy/cell_ (2.14) = 2.65, *p* < 0.02) (Figure 4).

### 3.3 Increased basal *Ucn1* mRNA expression was detected in the EWcp of TRPA1 KO mice

**FIGURE 5:**
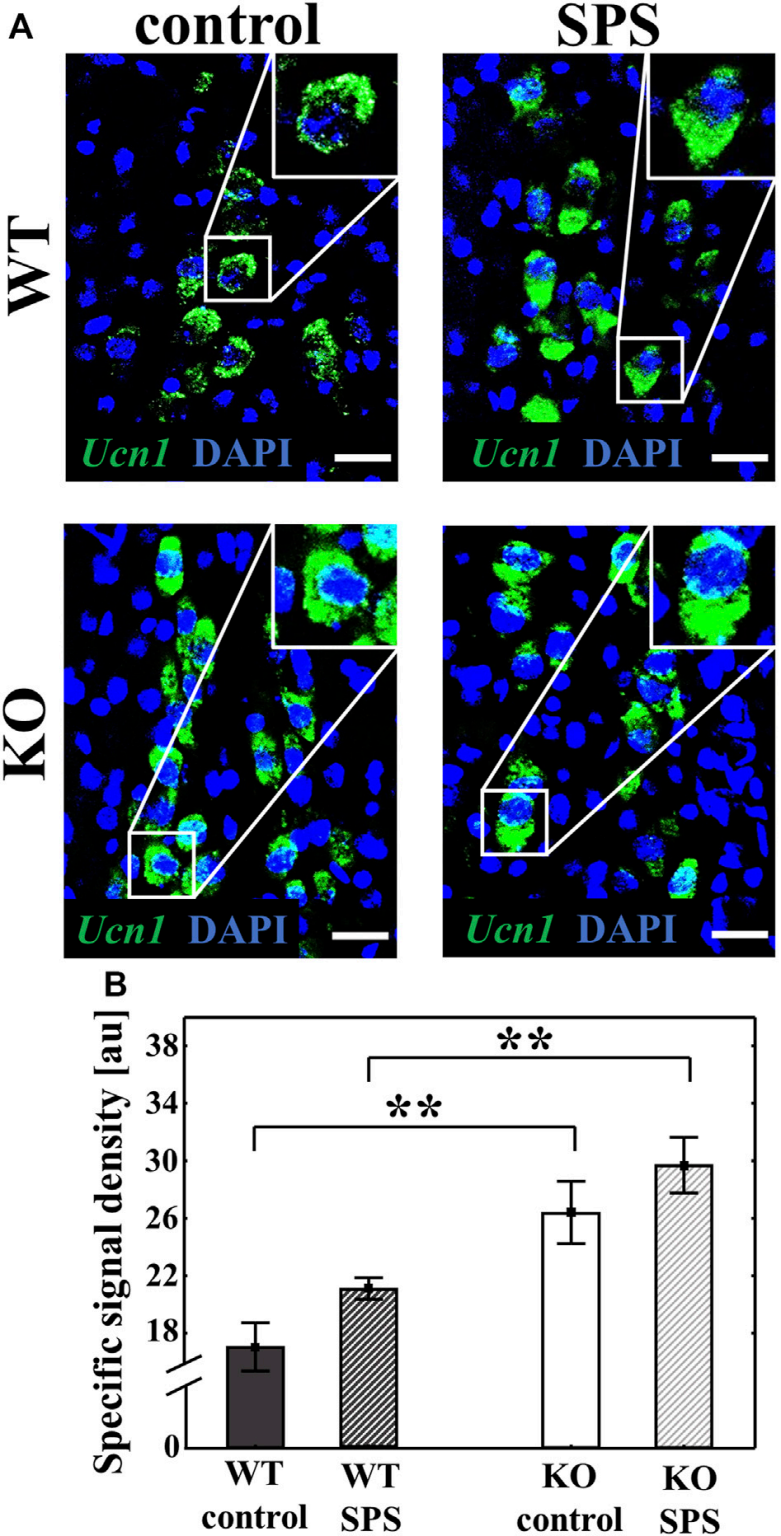
Alteration of the urocortin 1 (*Ucn1*) mRNA expression in the centrally projecting Edinger-Westphal nucleus (EWcp) upon single prolonged stress (SPS). Representative confocal images of the *Ucn1* (green) mRNA expression in the EWcp neurons, visualized by RNAscope *in situ* hybridization. Sections were counterstained with 4′,6-diamidino-2-phenylindole (DAPI) (blue) for nuclei. Scale bars: 25 µm **(A)**. Columns represent the specific signal density of *Ucn1* mRNA/neuron **(B)** (two-way ANOVA followed by Tukey’s *post-hoc* test; **p < 0.01; n = 8/group). au: arbitrary unit. WT: wild type, KO: knockout.

RNAscope ISH was used to measure the *Ucn1* mRNA expression in the EWcp of WT and KO mice. Both basal and SPS-induced *Ucn1* expression was significantly elevated in the KO animals compared to the WT counterparts with a strong main effect of the genotype (F_genotype_ (1.22) = 30.70, *p* < 0.01) and the stress (F_SPS_ (1.22) = 4.99, *p* < 0.04) without interaction. Although stress has a main effect in ANOVA test, upon Tukey’s *post hoc* comparison there was no significant difference between controls and SPS-treated groups (both p_WT_ and p_KO_ > 0.30). (Figure 5).

### 3.4 TRPA1 KO mice do not show elevated UCN1 peptide content of EWcp neurons upon SPS

**FIGURE 6:**
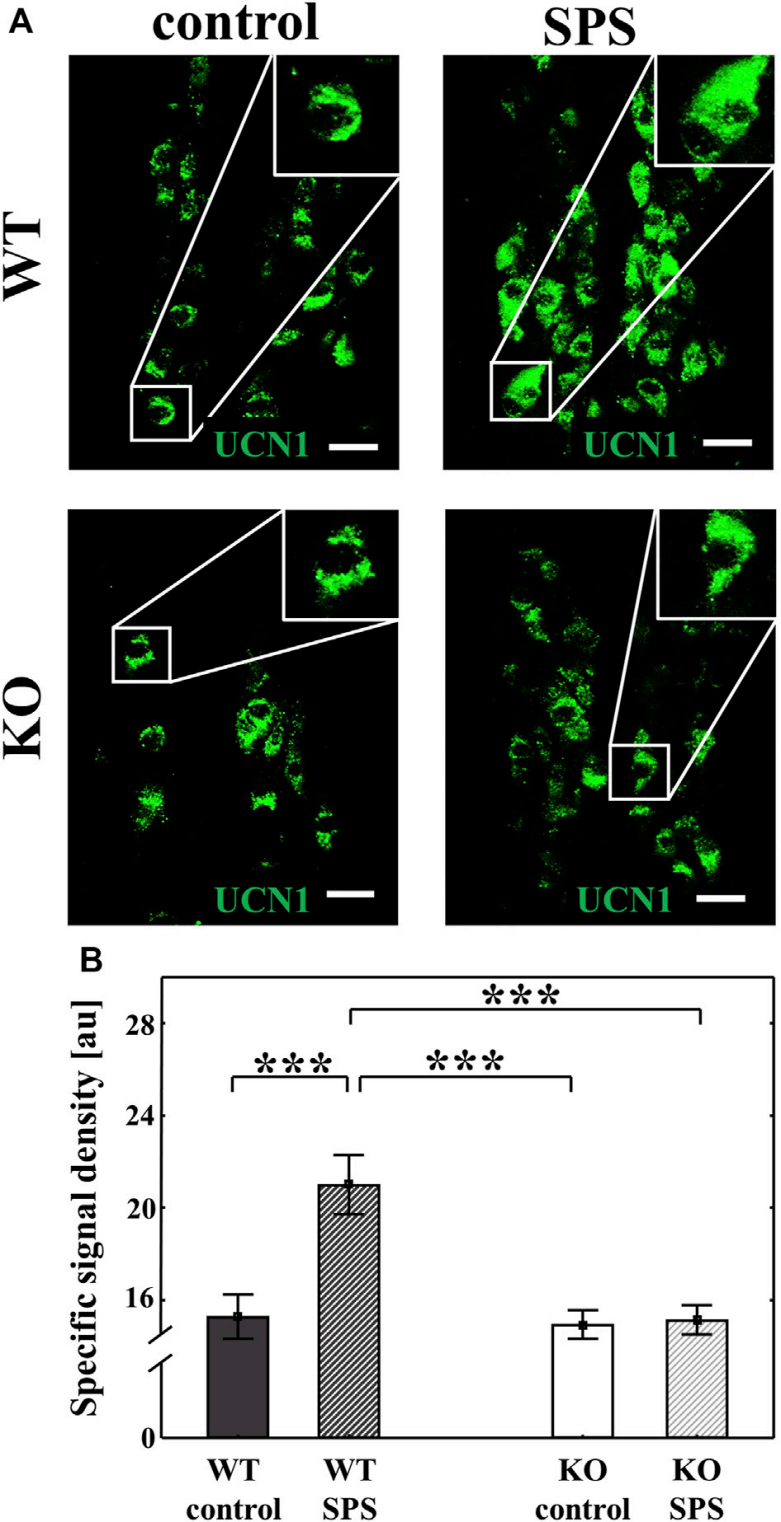
Effects of single prolonged stress (SPS) on the urocortin 1 (UCN1) peptide content of the centrally projecting Edinger-Westphal nucleus (EWcp) neurons. Representative confocal images of the UCN1 (green) immunofluorescence in the EWcp neurons. Scale bars: 25 µm **(A)**. Columns represent the specific signal density of urocortin 1 peptide/neuron **(B)** (two-way ANOVA followed by Tukey’s *post-hoc* test; ***p < 0.001; n = 8/group). au: arbitrary unit, WT: wild type, KO: knockout.

We performed immunofluorescence staining to detect the UCN1 in the EWcp neurons. UCN1 peptide content was significantly increased in WT animals upon SPS (F_SPS_ (1.28) = 10.29, *p* < 0.01), however, no SPS-induced changes were observed in KO mice (F_genotype_ (1.28) = 11.36, *p* < 0.01), which was supported with a strong interaction in ANOVA (F_interaction_ (1.28) = 8.95, *p* < 0.01, upon Tukey’s *post hoc* comparison both p_WT_ and p_KO_ < 0.01) (Figure 6).

## 4 Discussion

In mice, the PTSD model SPS diminished the *Trpa1* mRNA content in the EWcp with a concomitant increase in its UCN1 content (see also Supplementary Figure S1 for independent replication). Additionally, the SPS-induced PTSD-like behavioral symptoms were diminished in the lack of TRPA1, together with a prevention of EWcp/UCN1 protein increase. Controversially, UCN1 mRNA of the EWcp was higher in KO than WT, independently from stress.

The behavioral results proved the reliability of the SPS as PTSD model as the WT mice showed higher immobility during both the FST and restraint tests, characteristic for the PTSD (Lisieski et al., 2018; Verbitsky et al., 2020). The increased immobility during both tests were supported by findings of other research groups (Yamamoto et al., 2009; Serova et al., 2013, 2014; Sabban et al., 2015). Moreover, our present observation in FST that the lack of TPRA1 prevented stress-induced behavioral changes is consistent with our previous findings in the CVMS model (Kormos et al., 2022). These behavioral observations suggest a functional role of TRPA1 in stress adaptation.

As *Trpa1* mRNA occurred in the EWcp in the greatest amount, next we focused on this area. First, we replicated our recent findings (Kormos et al., 2022; Al-Omari et al., unpublished), that exclusively UCN1 positive cells express *Trpa1* in the mouse EWcp (Supplementary Figure S2). *Trpa1* mRNA expression was significantly reduced upon SPS in WT mice, which is in full agreement with our previous results in the CVMS model (Kormos et al., 2022). The high translational value and the relevance of TRPA1 in stress (mal)adaptation is further supported by our findings in EWcp samples of suicide victims where the *TRPA1* mRNA was also downregulated (Kormos et al., 2022).

As we have observed that the downregulation of *Trpa1* is a common phenomenon in these stress models, the question arises as to what might be behind this change. One plausible explanation from the pharmacological point of view may be that the action of an agonist may lead to the downregulation of its target (Rang et al., 2018). TRPA1 ion channels are known to be activated by glia-derived (Hori and Kim, 2019; Oroian et al., 2021) reactive free radicals including lipid peroxidation products and prostanoids (Logashina et al., 2019; Talavera et al., 2020), mediators released during oxidative stress, neuroinflammation and importantly, in PTSD (Yehuda, 1998; Kasckow et al., 2001; Rohleder et al., 2010; Ozdemir et al., 2012; Aschbacher et al., 2013; Bulut et al., 2013; Miller et al., 2015, 2018; Atli et al., 2016; Stoop, 2016; Hori and Kim, 2019; Oroian et al., 2021). Further molecular pharmacological studies are required to identify the exact role of inflammatory mediators in the regulation of *Trpa1* expression in the EWcp. In the lack of a widely trusted TRPA1 antibody, our present work provides only findings at mRNA level regarding TRPA1. Importantly, most recently we proved the presence of functionally active TRPA1 in EWcp/UCN1 cells by electrophysiological tools (Al-Omari et al. unpublished).

The modulatory role of TRPA1 in the control of UCN1 cells is further supported by our findings, that in the absence of the functional receptor, neither the *Ucn1* mRNA nor the UCN1 peptide content of the cells increased in the SPS model. Interestingly, this was in line with unchanged FST immobility time in SPS-exposed KO mice. This altered stress response might be explained by the reduced adaptation capacity of the UCN1 cells in KO mice as shown both by higher basal *Ucn1* mRNA expression level compared to the WT counterparts, and by absence of response to SPS. Interestingly, we observed the same basal difference between the genotypes in CVMS model (Kormos et al., 2022). We might assume that TRPA1 will also affect the release of UCN1 and thereby stress adaptation (Kozicz et al., 2001; Gaszner and Kozicz, 2003; Gaszner and Kozicz, 2003; Gaszner et al., 2007, 2012; Rouwette et al., 2011; Kormos and Gaszner, 2013; Kormos et al., 2022; Ujvári et al., 2022). Although we do not provide experimental evidence for this in the present study, it is already known that activation of the TRPA1 cation channel may increase the neuropeptide release (e.g., UCN1) *via* elevated intracellular calcium level (Denner et al., 2017; Casello et al., 2022). We might assume that the stress-induced decrease in *Trpa1* expression is aimed to compensate the exaggerated UCN1 release and thereby the behavioral responses. However, further investigations are needed to confirm this theory. Moreover, there was a discrepancy between the low EWcp/UCN1 peptide content of the TRPA1 KO mice despite their high *Ucn1* mRNA levels, which may be explained by the altered dynamics of UCN1 peptide release. It is supported by the previously mentioned role of TRPA1 in the intracellular calcium homeostasis, as well as by its possible developmental influence (Asai et al., 2010). However, we cannot exclude that other cation channels expressed in the EWcp (Li and Ryabinin, 2022), may have compensated for the lack of TRPA1 in KO mice.

Finally, it is well-known that PTSD may show co-morbidity with enhanced pain sensitivity (Gibson, 2012) and somatoform disorders (Afari et al., 2014; Egle et al., 2016). Interestingly, epigenetic modifications on the promoter of *TRPA1* gene in human leukocytes were found in these conditions (Achenbach et al., 2019). Since the epigenetic profile of a gene promoter may be similar in both the peripheral and central nervous system (Davies et al., 2012) it is plausible that these epigenetic changes may also affect TRPA1 on EWcp/UCN1 neurons. We earlier found that maternal deprivation blunts the stress responsivity of UCN1 neurons in the rat EWcp (Gaszner et al., 2009). Consistently, early life stress in mice caused epigenetic modifications of histone H3 acetylation in EWcp/UCN1 cells if superimposed with CVMS (Gaszner et al., 2022). These data raise the possibility that the EWcp/UCN1 neurons may contribute not only to the development of major depression (Kormos and Gaszner, 2013; Farkas et al., 2017)) but also may underlie PTSD, a disease that share similar epigenetic pathobiological mechanisms (Blacker et al., 2019).

## 5 Conclusion and future perspectives

**FIGURE 7:**
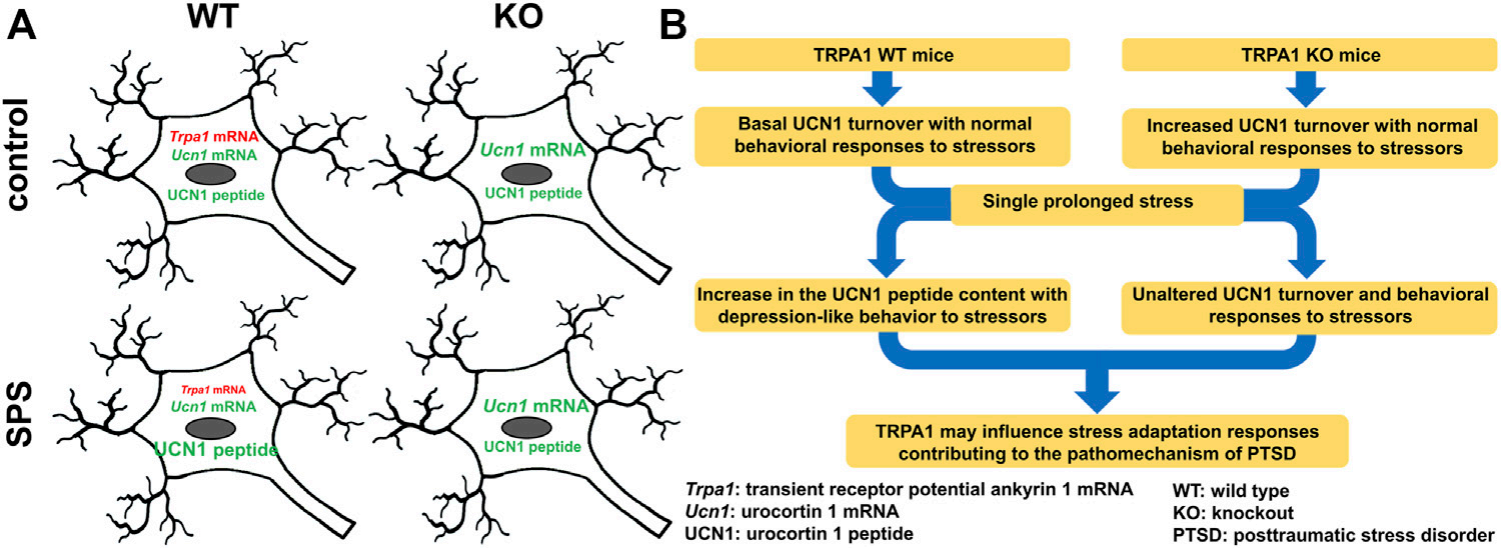
Functional-morphological **(A)** and graphical **(B)** summary of changes in the urocortinergic centrally projecting Edinger-Westphal nucleus in the single prolonged stress (SPS) model of PTSD in TRPA1 WT and KO mice.

Decreased *Trpa1* mRNA expression in the PTSD model was associated with increased neuronal UCN1 peptide content in the EWcp. This suggests the involvement of this cation channel in stress (mal)adaptation contributing to the pathomechanism of PTSD (Figure 7). In our ongoing research we examine the recruitment of EWcp/TRPA1/UCN1 neurons in models of PTSD in contexts of neuroinflammation and epigenetics.

## Data Availability

The raw data supporting the conclusion of this article will be made available by the authors, without undue reservation.
