# Supplementary material for: Transient receptor potential ankyrin 1 ion channel expressed by the Edinger-Westphal nucleus contributes to stress adaptation in murine model of posttraumatic stress disorder
Source: Front Cell Dev Biol. 2022 Dec 6;10:1059073. doi: 10.3389/fcell.2022.1059073 (PMC9763580; doi:10.3389/fcell.2022.1059073)
Supplement: Supplementary file 1 [file Presentation1.pdf]

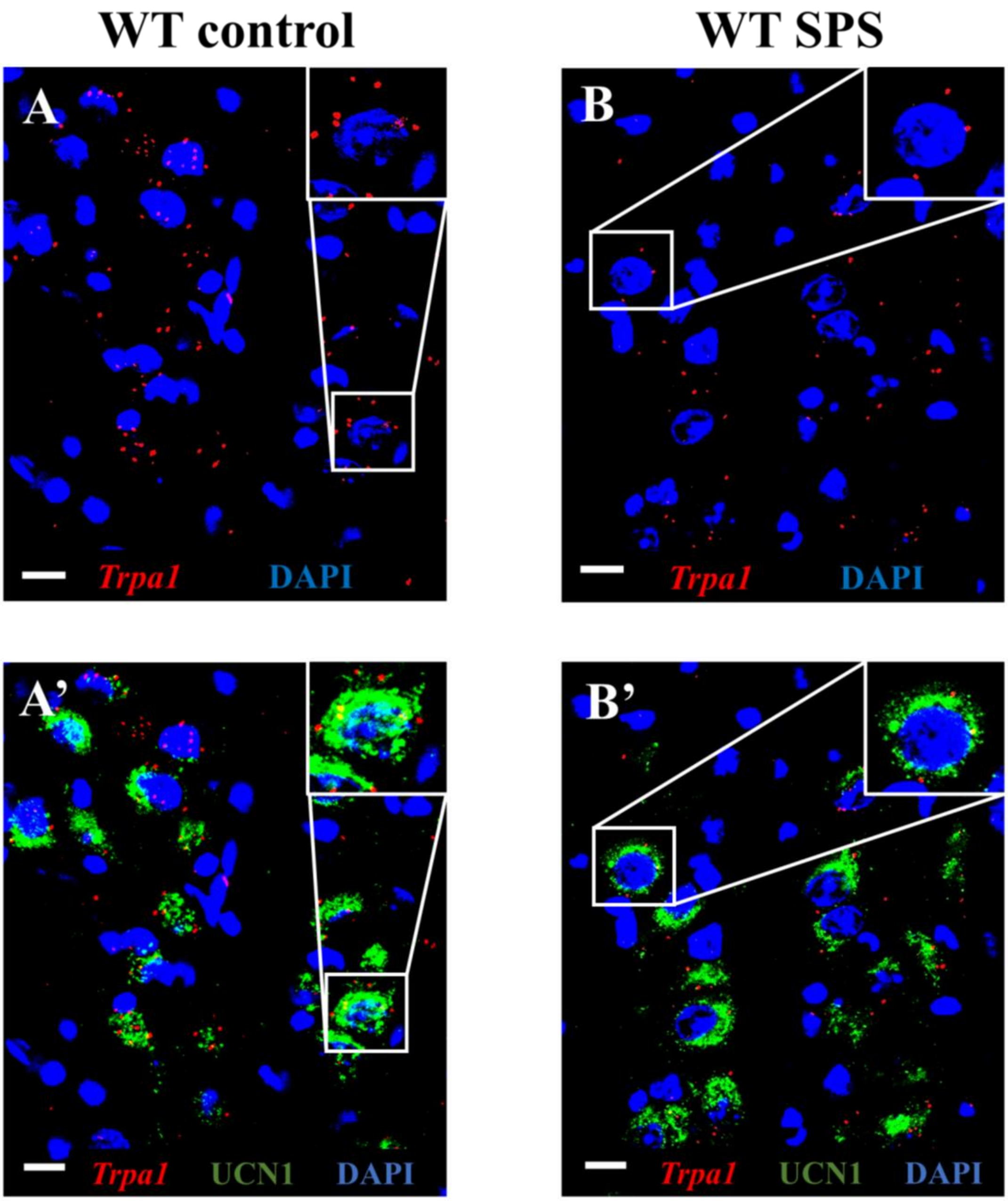

Supplementary Figure 1. Effect of single prolonged stress (SPS) on the *Trpa1* expression in the centrally projecting Edinger-Westphal nucleus (EWcp). Representative image of the *Trpa1* mRNA expression in the urocortinergic EWcp neurons of wild type (WT) mice **(A-B)**. The colocalization of *Trpa1* mRNA with the urocortin 1 (UCN1) immunostaining **(A'-B')**. Red dots represent *Trpa1* mRNA copies while cell nuclei were counterstained with DAPI (blue). Green color shows the immunosignal of UCN1 **(A'-B')**. Bars: 25 µm

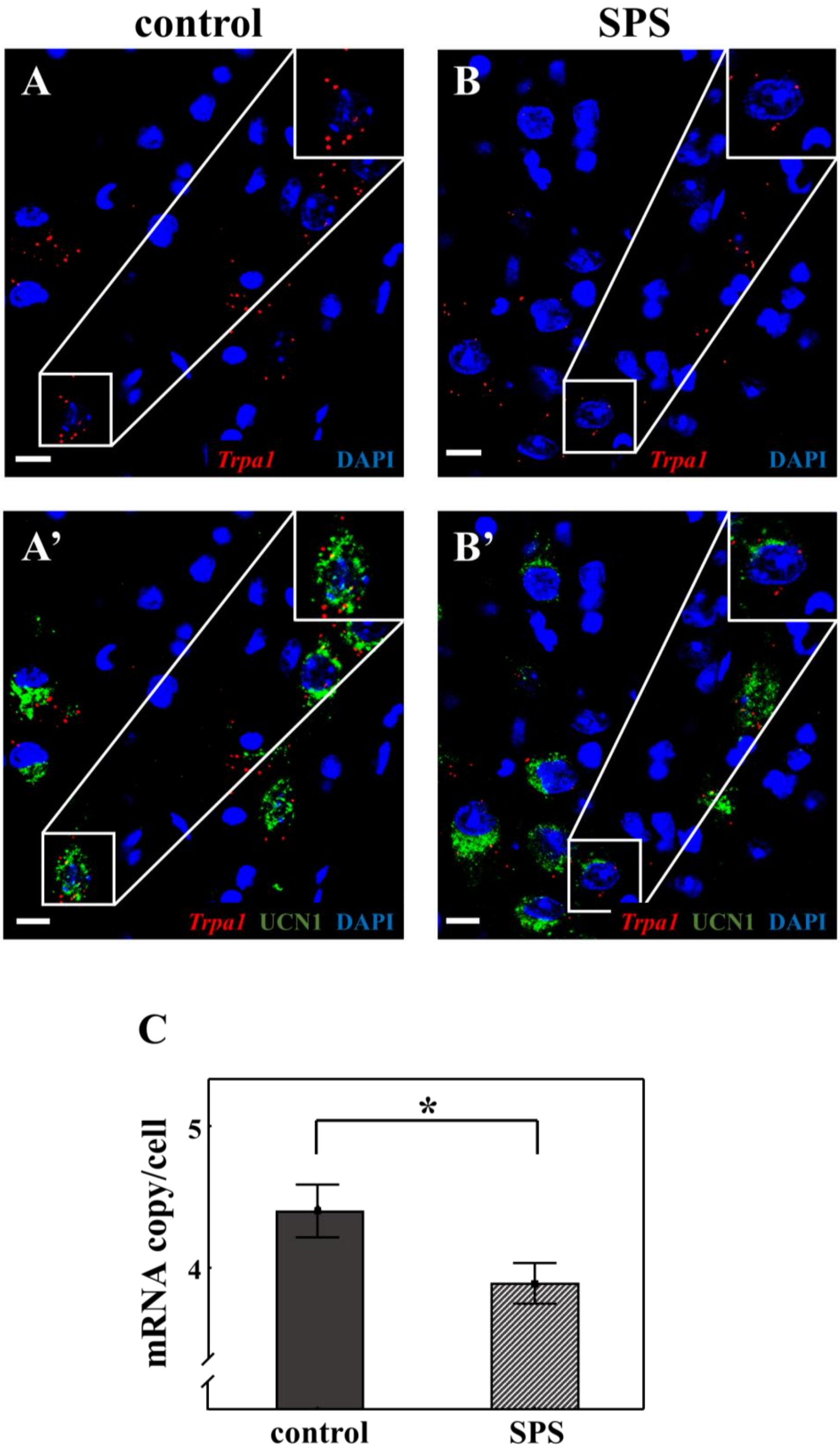

Supplementary Figure 2. Effect of single prolonged stress (SPS) on the *Trpa1* expression in the centrally projecting Edinger-Westphal nucleus (EWcp). Representative image of the *Trpa1* mRNA expression in the urocortinergic EWcp neurons of C57BL/6 mice **(A-B)**. The colocalization of *Trpa1* mRNA with the urocortin 1 (UCN1) immunostaining **(A'-B')**. Red dots represent *Trpa1* mRNA copies while cell nuclei were counterstained with DAPI (blue). Green color shows the immunosignal of UCN1 **(A'-B')**. Bars: 25 µm. Statistical analysis established significantly decreased amount of *Trpa1* mRNA in the EWcp after SPS (Student's *t*-test; *$p < 0.05$; n = 7/groups) **(C)**.
